# Matrix Stiffness Directs Stemness Signatures in Breast Cancer

**DOI:** 10.1002/adhm.71371

**Published:** 2026-06-22

**Authors:** Chantal Kopecky, Elvis Pandzic, Sean Porazinski, J Justin Gooding, Kristopher A Kilian

**Affiliations:** ^1^ School of Chemistry, Australian Centre for NanoMedicine, Faculty of Science UNSW Sydney Sydney Australia; ^2^ Katharina Gaus Light Microscopy Facility, Mark Wainwright Analytical Centre UNSW Sydney Sydney Australia; ^3^ Inventia Life Science Sydney Australia; ^4^ School of Materials Science & Engineering Faculty of Science UNSW Sydney Sydney Australia

**Keywords:** 3D cancer models, bioprinting, breast cancer, cancer stemness, matrix stiffness, phenotypic plasticity

## Abstract

Phenotypic plasticity contributes to tumor progression and metastasis, with the tumor microenvironment playing a central role through dynamic cues such as extracellular matrix stiffness. In this study, 2D and 3D in vitro breast cancer models were developed to investigate how ECM mechanics regulate cancer cell behavior. Hydrogel micropatterning enabled the mimicry of spatial confinement and stiffness in 2D microtumors, while drop‐on‐demand bioprinting facilitated the fabrication of mechanically tuneable 3D matrices. Phenotypic characterisation was conducted using immunofluorescence staining for molecular markers of plasticity and stemness, and drug resistance was assessed with doxorubicin and enzalutamide, the latter chosen for its emerging relevance in targeting stem‐like cancer cell populations. Soft matrices promoted stem‐like phenotypes, elevated ALDH1 expression, and enhanced drug resistance, whereas stiff matrices maintained a more differentiated profile. CD44 isoform expression was stiffness‐dependent, with the CD44 standard isoform enriched in soft matrices and the CD44 variant 9 isoform enriched in stiff matrices. The 3D matrices reproduced the mechanical regulation observed in 2D, providing a physiologically relevant platform for high‐throughput investigation of biomechanics‐driven cancer progression. These findings highlight the role of matrix stiffness in driving breast cancer phenotypic heterogeneity and support the application of microengineered synthetic matrices for studying metastasis and drug resistance.

## Graphical abstract

We built lab‐grown breast cancer models to study how the stiffness of the tumor's surroundings influences cancer behavior. Softer environments encouraged more stem‐like, drug‐resistant cells, while stiffer ones kept cells more differentiated. These findings show that tissue mechanics drive cancer diversity and drug resistance, offering new insights for studying and treating metastasis.

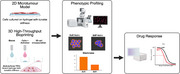

## 1 Introduction

Cellular plasticity is increasingly recognized as a hallmark of cancer [1], enabling tumor cells to shift phenotypes in response to microenvironmental changes, which contributes to progression, metastasis, and treatment resistance [2, 3]. Despite its central role in tumor adaptation, plasticity remains a largely unexplored therapeutic target [4].

A key indication of plasticity in solid tumors is the emergence and persistence of cancer stem cells (CSCs), a subpopulation with self‐renewing, tumorigenic, and therapy‐resistant capabilities [5, 6]. CSCs drive tumor initiation, sustain growth, and are often responsible for relapse post‐treatment [7, 8]. Their adaptability and survival mechanisms render them resistant to conventional therapies, leading to poor long‐term outcomes. Importantly, CSCs are dynamic and non‐stem tumor cells can acquire stem‐like traits under stress, further contributing to heterogeneity and treatment failure [9].

In breast cancer, breast cancer stem and progenitor cells are central to tumor initiation, progression, and clinical response [10, 11, 12]. Breast cancer CSCs (BCSCs) are characterized by markers such as CD44^+^, ALDH1^hi^, CD133^+^, and are associated with high plasticity and poor prognosis [13, 14, 15]. Some BCSCs occupy a hybrid epithelial/mesenchymal state, enhancing tumorigenicity and resistance to chemotherapy [16, 17]. As current therapies typically do not eliminate BCSCs [18], there is a critical need for approaches targeting this population specifically.

Biomechanical cues including matrix stiffness, geometry, and extracellular matrix composition have emerged as powerful regulators of cancer cell fate, influencing gene expression, morphology, stemness, and invasiveness [19, 20, 21, 22, 23, 24]. For example, triple‐negative breast cancer (TNBC) cells primed in soft matrix conditions have been shown to display enhanced survival under stress, anchorage‐independent growth, and increased metastatic potential, in contrast to the more proliferative, invasive behavior seen in stiff matrices [25]. These findings suggest that biomechanical priming at the primary tumor site may predefine metastatic behavior, especially in aggressive subtypes.

However, many existing experimental models fail to capture the complexity of cancer plasticity. Traditional 2D cultures often lack the spatial and mechanical context of the in vivo tumor environment [26, 27], limiting their ability to replicate CSC behavior. This contributes to translational failure, with only ∼10% of candidate cancer drugs succeeding in clinical trials [28].

To overcome these limitations, 3D bioengineered and bioprinted tumor models are gaining traction. These platforms enable the integration of defined matrix components, stiffness gradients, and tissue‐like architecture, offering enhanced biomimicry of the tumor microenvironment. They allow for precise studies of CSC dynamics, phenotypic plasticity, and drug responses [29, 30, 31, 32]. In addition to improving complexity, such models support high‐throughput screening, reduce animal use, and enable personalized treatment strategies [33].

Together, understanding how biomechanics regulate CSC plasticity and integrating this knowledge with advanced 3D model systems, offers a promising pathway toward more effective cancer therapies and improved translational success.

Here, we applied both 2D and 3D in vitro models incorporating defined biomechanical tumor microenvironment properties to demonstrate how matrix stiffness governs phenotypic outcomes in TNBC. We found that stiffness‐dependent changes not only regulate distinct cellular profiles but also modulate drug response and resistance patterns, underscoring the importance of biomechanical context in therapeutic evaluation and application of personalised medicine approaches.

## 2 Results

### 2.1 Matrix Mechanics Drive Stemness and Migratory Phenotypes in Breast Cancer in 2D

**FIGURE 1:**
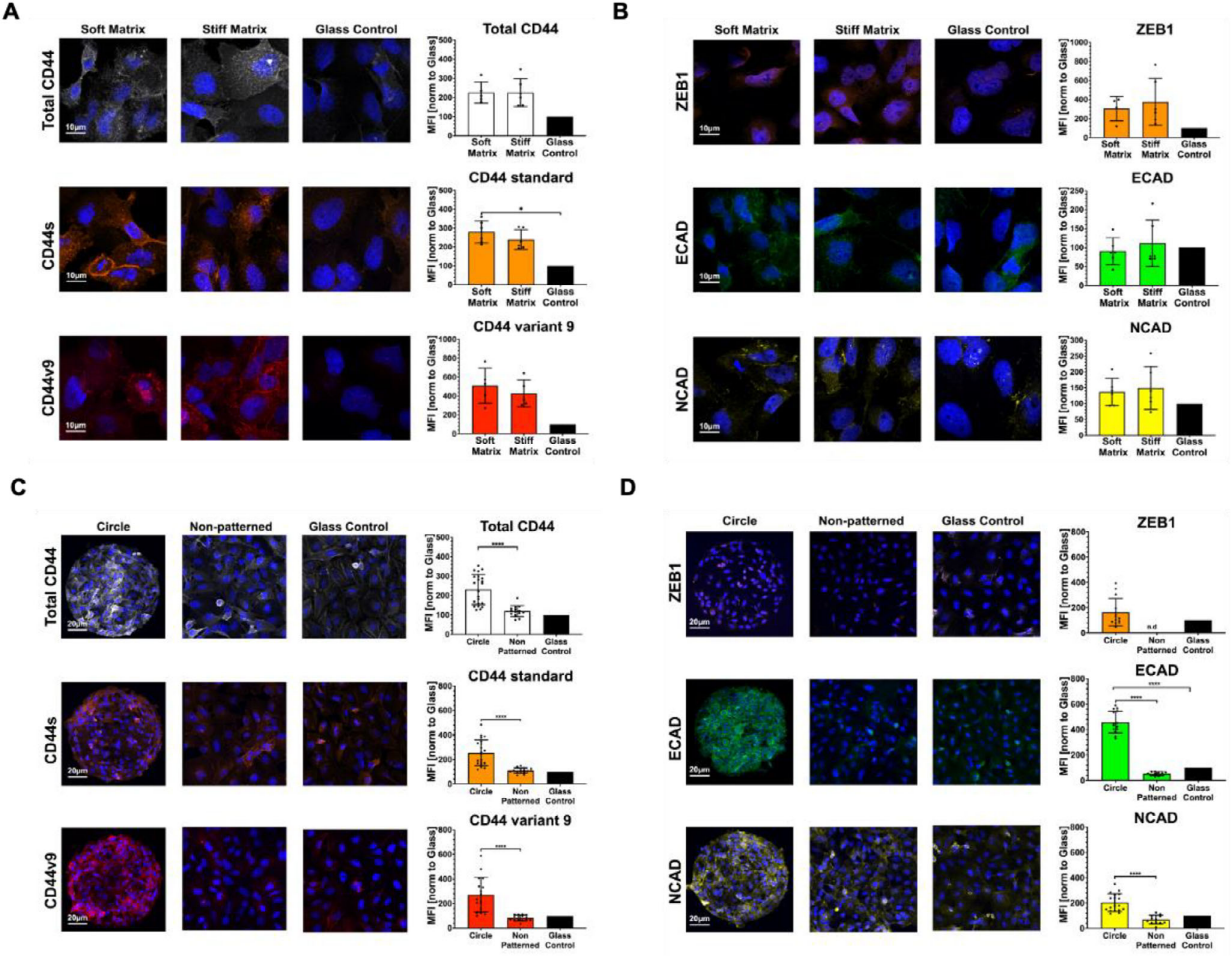
Phenotypic profiling of 2D breast cancer microtumors immunostained for stemness (CD44 total, CD44s, CD44v9) and migratory markers (ZEB1, ECAD, NCAD). (A, B) HCC38 cells were cultured for 5 d on non‐patterned hydrogel substrates (1 and 100 kPa) and glass (Control). (C, D) HCC38 cells were cultured for 5d on circle‐patterned, non‐patterned hydrogels (10 kPa) and glass (Control). Fluorescent intensities are expressed as % of glass control. *p < 0.05; ****p < 0.0001.

To investigate the effect of matrix stiffness on breast cancer phenotype, we used a 2D platform where oxidized matrix protein is covalently bound to hydrazine‐treated polyacrylamide hydrogels, with stiffness tuned from <1‐100 kPa by varying crosslinker concentration. HCC38 cells were cultured on soft matrix (1 kPa) or stiff matrix (100 kPa) fibronectin‐coated polyacrylamide substrates as described previously [19]. After 5 days, we fixed and immunostained the cultures to evaluate the expression of stemness and migration markers. We found that CD44 expression (isoforms and total) was upregulated comparably on both stiffnesses compared to cells grown on glass (Ctr) (Figure 1A). Similarly, EMT markers ZEB1, E‐cadherin and N‐cadherin expression increased in cells adherent to compliant matrix coated conditions (Figure 1B).

Next, since cells in most tissue are spatially confined, we were examined the role of confinement on phenotypic profiles. To confine cells to regions across the polyacrylamide substrate, we used soft lithography with a structured polydimethylsiloxane stamp, where the matrix protein was deposited in periodic spaced islands of 500 µm^2^. We found that cells grown on circular patterns for 5 days showed a marked upregulation of all CD44 markers compared to those grown on non‐specific patterns and to controls (Figure 1C). The EMT markers ZEB1, E‐cadherin and N‐cadherin were significantly upregulated in cells grown on the circle patterns compared to those experiencing no spatial confinement (Figure 1D). These results demonstrate how confinement can influence the phenotype of cells. However, in this case there were no obvious differences on account of matrix stiffness.

### 2.2 Matrix Stiffness Differentially Regulates Phenotypic Plasticity in 3D Bioprinted Breast Cancer Models

**FIGURE 2:**
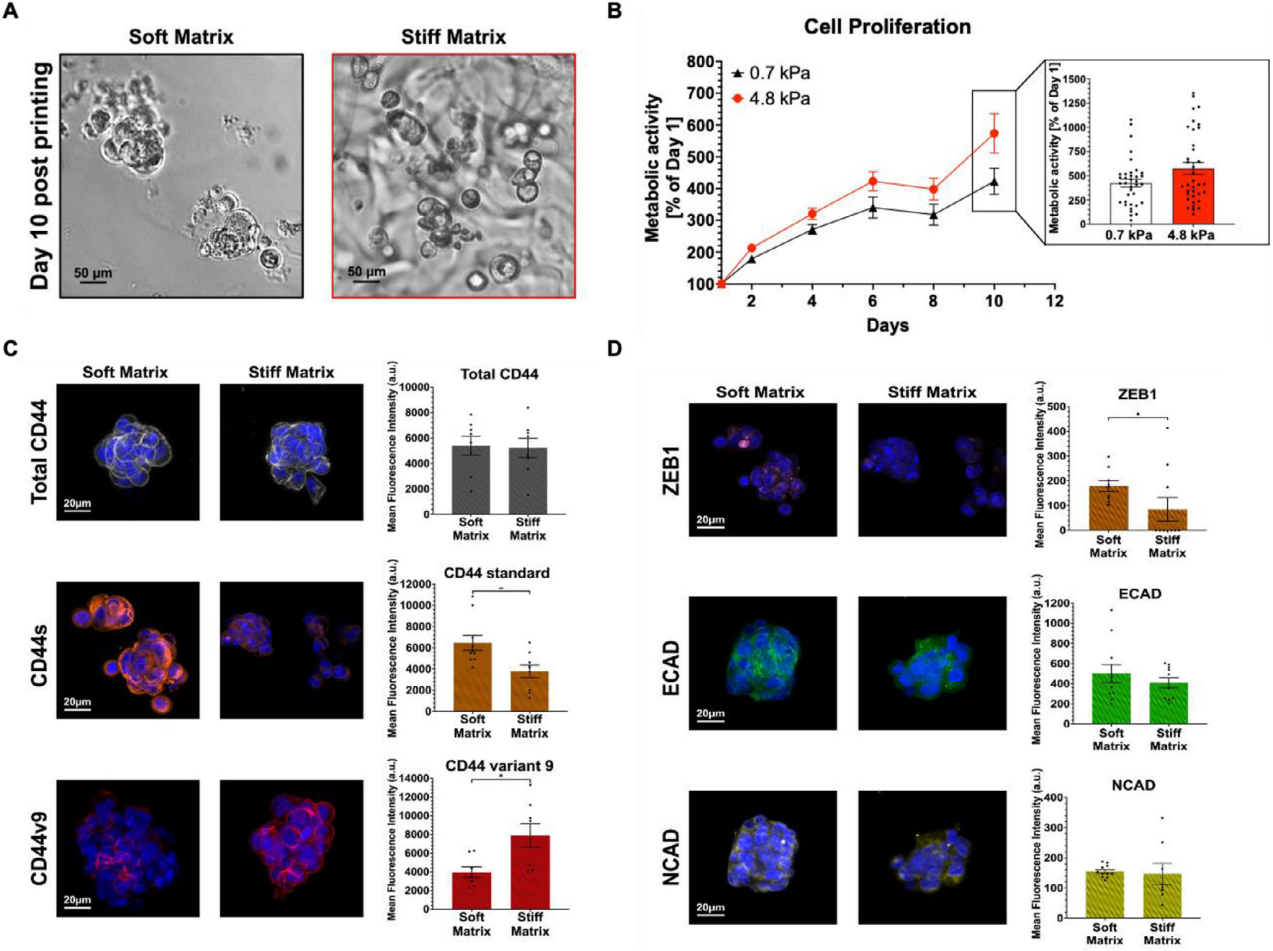
Morphological and phenotypic characterisation of 3D bioprinted breast cancer models after 10 d of culture. (A) Representative brightfield images of HCC38 spheroid morphologies cultured in soft (0.7 kPa) and stiff (4.8 kPa) matrices. (B) Cell proliferation monitored over 10 d via metabolic assays (AlamarBlue, measured every 2 d). (C) HCC38 tumoroids were immunostained for indicated stemness markers and (D) migratory markers.

Seeking a more physiologically representative and complex in vitro model, we utilised the RASTRUM 3D bioprinting technology from Inventia Life Sciences, which allows for reproducible and high throughput generation of biofunctional synthetic matrices with modular control over composition and stiffness. We used PEG‐based hydrogels with incorporated extracellular matrix peptides derived from laminin, fibronectin, collagen I, with stiffnesses of 0.7 kPa (soft matrix) and 4.8 kPa (stiff matrix) and matrix metalloprotease degradable peptides to allow migration and proliferation. We printed HCC38 cells within each matrix in situ and cultured them for up to 10 days. We found that cells grown in stiff matrix conditions formed slightly smaller cell clusters with an average of 12 cells/cluster vs. 15 cells/cluster in soft matrix conditions (Figure 2A and Figure S1A) and presented a similar overall sphericity (Figure S1B). Stiff matrix culture resulted in a higher growth rate compared to those exposed to soft matrices (Figure 2B). The cultures were fixed and immunostained at 10 days for a panel of molecular markers associated with stemness (CD44 isoforms), plasticity (ALDH1, CD133, ABCB1, Ki67) and EMT markers (ZEB1, E‐cadherin, N‐cadherin). Interestingly, the CD44 standard isoform showed significantly higher expression in cells cultured within soft matrix conditions. In contrast, cells in the stiff matrix culture showed a significant upregulation of the CD44 variant 9. These changes were not reflected in the total CD44 expression, which was comparable in both matrix conditions (Figure 2C). Expression of ALDH1, CD133 and ABCB1 was distinctly higher in cells exposed to the soft matrix, whereas the proliferation marker Ki67 tended to be more present in stiff matrix conditions (Figure S2), which aligns with the observed increase in proliferation (Figure 2B). The migratory characteristics indicated a specific EMT/MET profile with soft matrix culture resulting in higher expression of ZEB1, and comparable E‐cadherin and N‐cadherin levels (Figure 2D). Notably, inclusion of an intermediate matrix stiffness (3 kPa) revealed responses comparable to those at 4.8 kPa, indicating that the observed phenotypic trends are robust across this broader stiffness range (data not shown).

Since triple negative breast cancer is a highly heterogeneous disease, we also profiled a more widely used highly invasive cell line, the MDA‐MB‐231 population (Figure S3). While we observed some trends in phenotype markers as a function of matrix stiffness under the same culture conditions, these differences were modest and not statistically significant. It should be noted that the HCC38 cells are well known to demonstrate phenotype plasticity on account of culture conditions. These findings further support that mechanobiological responses are strongly context‐ and cell line‐dependent.

### 2.3 Soft Matrix Induces a Specific Stemlike Population That Elicits Higher Chemoresistance

**FIGURE 3:**
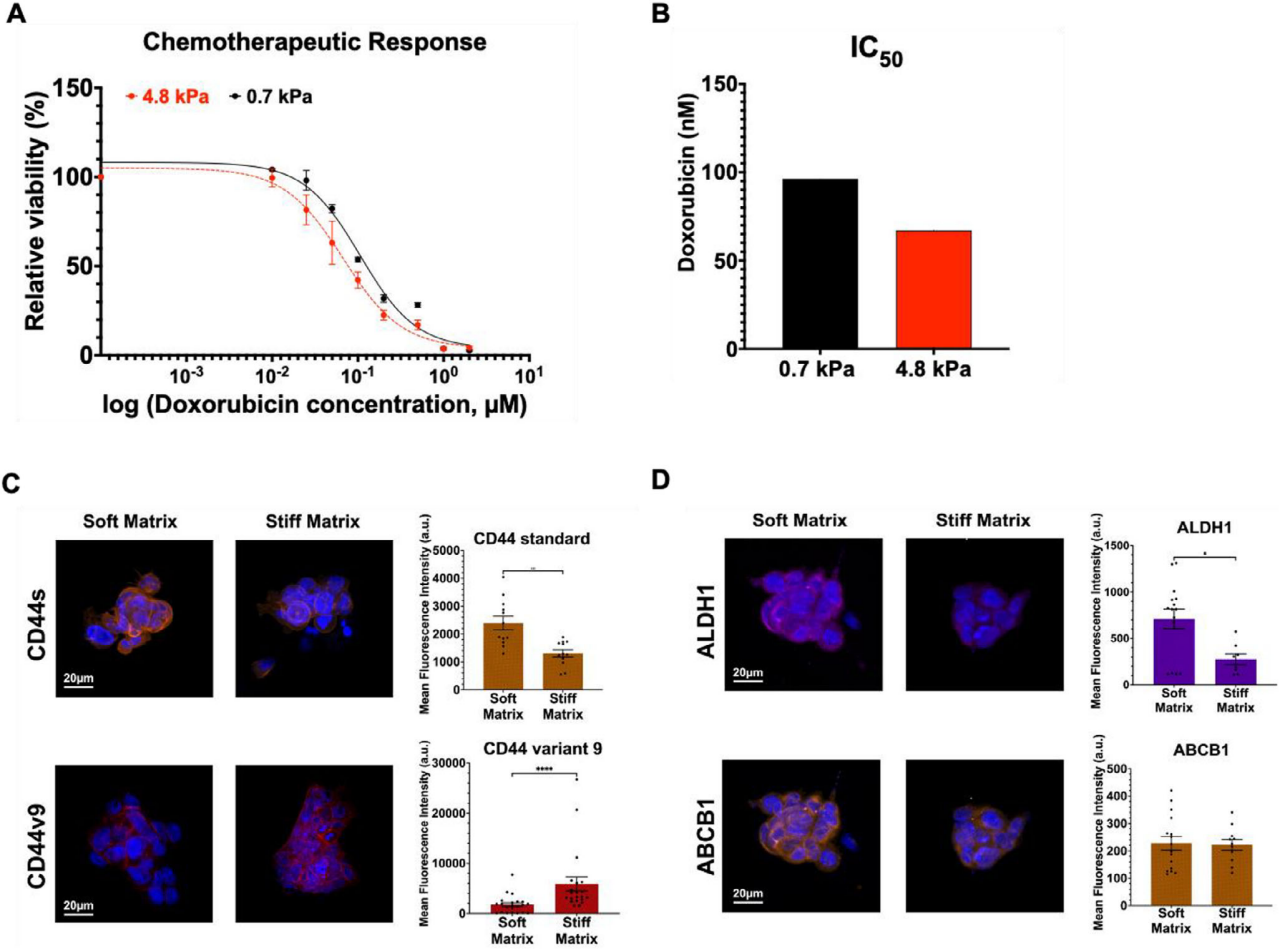
Chemotherapeutic response of 3D bioprinted breast cancer models. (A) Dose‐response curves of HCC38 tumoroids encapsulated for 5 days in soft (0.7 kPa) and stiff (4.8 kPa) matrices treated with various doxorubicin concentrations for 72 h, followed by CellTiter‐Glo viability assays. (B) Half maximal inhibitory concentrations (IC_50_) for different stiffness conditions. (C) Immunostaining for indicated stemness markers and (D) drug efflux transporters at the time of treatment (day 5 post‐printing).

Since the soft matrix conditions nurtured the appearance of a stem cell‐like population, which has been demonstrated to correspond to drug resistance characteristics in breast cancer [34], we next evaluated drug responses to the standard‐of‐care chemotherapeutic doxorubicin. Drug was added at day 5 of culture for a further 72 h across a concentration range spanning 0.01 – 2 µm with control cultures receiving medium only. The soft matrix conditions exhibited a significant difference in IC_50_, resulting in greater drug resistance compared to stiff matrices (Figure 3A,B). Further characterization revealed that cells cultured under soft conditions displayed a distinct stem‐like signature at the time of treatment, with significant upregulation of CD44s, while CD44v9 remained significantly elevated in the stiff matrix (Figure 3C). Notably, the CD44s^hi^ phenotype was accompanied by a marked increase in the expression of the stemness marker and drug efflux transporter ALDH1, whereas ABCB1 expression showed a small trend to upregulation in soft conditions (Figure 3D). Together, these results suggest that the increased drug resistance observed under soft matrix conditions is associated with upregulated drug efflux transporters and the maintenance of distinct stem‐like characteristics.

### 2.4 Anti‐Androgen Treatment Targets the Chemoresistant Stem Fraction in Breast Cancer

**FIGURE 4:**
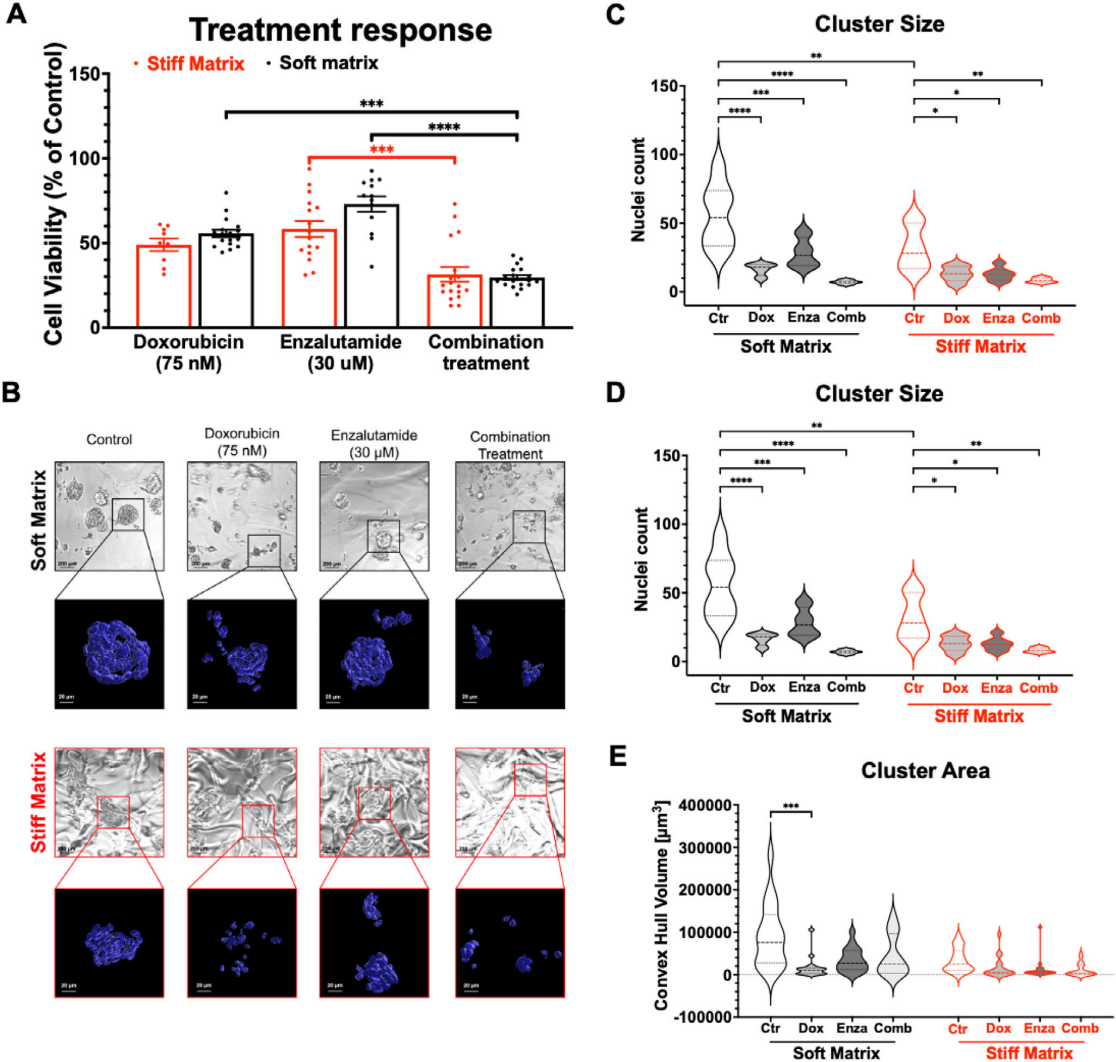
Novel treatment strategies successfully target breast cancer stem fractions induced by soft matrices. (A) HCC38 tumoroids cultured for 5 d in soft (0.7 kPa) and stiff (4.8 kPa) matrices were treated with indicated drugs for 72 h, followed by CellTiter‐Glo viability assays. (B) Morphological effects of drug treatments on 3D bioprinted breast cancer models. Representative brightfield and 3D rendered images of HCC38 tumoroids treated with indicated drugs. Quantitative analysis of (C) cluster size (determined by number of nuclei per cluster), (D) Intercellular distance (calculated by nearest neighbor distance of nuclei), and (E) Cluster volume (assessed by calculating 3D area metrics). Ctr, untreated control; Dox, doxorubicin; Enza, enzalutamide; Comb, combination treatment. *p < 0.05; **p < 0.01; ***p < 0.001; ****p < 0.0001.

Considering the stem‐like cellpopulation showed increased resistance to doxorubicin, we next assessed the response to enzalutamide, an anti‐androgen that has gained increasing attention for its potential in targeting stem cell‐like populations [35, 36]. While the HCC38 line is not a classic luminal androgen receptor (LAR) subtype TNBC model, there is evidence suggesting that androgen receptor (AR) expression and associated signalling contribute to phenotypic plasticity, and therapy resistance beyond LAR subtypes. Similar to treatment with doxorubicin, soft matrices fostered a subpopulation that showed some resistance to enzalutamide compared to cells in the stiffer matrices, albeit to a lesser degree (Figure S3A,B). Drug concentrations for combination treatment were selected based on the lower IC_50_ values (Figure 3B, Figure S4B) determined under stiff matrix conditions to ensure biologically relevant, sub‐maximal dosing. Notably, combination treatment with doxorubicin and enzalutamide demonstrated evidence for synergy, significantly reducing cell viability compared to either agent alone (Figure 4A).

Evaluation of post‐treatment cluster morphology revealed distinct responses to each drug (Figure 4B). Doxorubicin disrupted cell clusters, resulting in dispersed cell populations, while enzalutamide reduced cluster size but clusters retained some degree of aggregation. In contrast, the combination therapy was highly effective in both soft and stiff matrices, resulting in a near‐complete loss of viable cells and leaving behind only single or sparsely distributed cells with fragmented nuclei, indicative of widespread cell death (Figure 4C–E). These morphological effects were consistent across matrix stiffnesses, suggesting that the structural impact of treatment is largely independent of mechanical context.

Immunofluorescence staining revealed that doxorubicin treatment, both alone and in combination with enzalutamide, reduced cell proliferation as indicated by decreased Ki67+ staining. In contrast, enzalutamide alone had no significant effect on proliferation (Figure S5A). Single‐agent treatment of cells in soft matrix conditions further led to a reduction in CD44s expression (doxorubicin: ∼20%; enzalutamide: ∼40%, Figure S4B), whereas combination treatment unexpectedly restored CD44s levels to those seen in untreated controls, possibly reflecting adaptation and a more aggressive phenotype in the few surviving cells. Assessment of drug efflux transporters ALDH1 and ABCB1 highlighted this adaptive response. In soft matrices, ALDH1 and ABCB1 levels decreased following single‐agent treatment but were upregulated under combination treatment, suggesting a compensatory activation of efflux mechanisms. Conversely, in stiff matrices, all drug treatments led to increased expression of ALDH1 (Figure S5C,D).

Together, these findings demonstrate that while matrix softness promotes partial resistance to single‐agent therapies, combined treatment can overcome this effect, albeit with evidence of adaptive survival mechanisms that may contribute to long‐term resistance.

## 3 Discussion

The tumor microenvironment is increasingly recognized as a critical regulator of cancer progression, phenotypic plasticity, and therapeutic resistance. In this study, we demonstrate that matrix stiffness — a key biomechanical feature of the extracellular matrix — systematically directs phenotypic outcomes in breast cancer models and modulates drug sensitivity. Across both 2D and 3D model systems, our data show that soft matrices selectively enrich for stem‐like, therapy‐resistant cancer cell populations, reinforcing the concept that mechanical cues not only reflect but actively sculpt cancer plasticity during tumor development.

Using a 2D microtumor model, we show that substrate stiffness and spatial confinement cooperatively modulate cancer cell behavior. Our findings suggest that even in simplified models, cells are highly responsive to mechanical and architectural features of their environment, with curvature and mechanical stress gradients driving local reprogramming and heterogeneity. This underscores the idea that physical constraints may serve as early organizers of tumor phenotype [37].

To better reflect the architectural and mechanical complexity of the in vivo tumor microenvironment, we employed a 3D bioprinting platform to create three‐dimensional confined tumorspheres in matrices with defined composition and stiffness. Within these environments, we observed that softer matrices favored slightly larger, less proliferative cell clusters enriched for plasticity markers such as CD44s, ALDH1, and CD133, consistent with a stem‐like and migration/invasion‐inducing phenotype [38, 39, 40]. In contrast, stiffer matrices promoted smaller, more proliferative clusters, which showed increased expression of CD44v9 and Ki67 indicative of a distinct cancer cell subset potentially associated with populations primarily found at secondary outgrowth sites [41, 42]. These divergent behaviors reflect the context‐specific influence of biomechanical signals on cancer cell identity and indicate that softer metastatic niches (e.g., lung, brain, liver) may support plastic and chemoresistant cells [43, 44, 45].

Importantly, our findings advance the current understanding of dynamic phenotypic changes, offering new insights into how mechanical cues shape cancer cell behavior and potentially drive disease progression. Understanding the complex mechanical heterogeneity of the primary tumor, and how this influences cancer cell adaptivity at different stages of the metastatic cascade, is crucial for designing effective therapies. Our results, along with those of others, reveal that mechanical conditioning, and in particular soft environments can induce distinct cellular reprogramming and thus, influence the metastatic behavior and therapeutic response of cancer cells [46, 47]. This concept of stiffness priming is particularly relevant in aggressive subtypes such as triple‐negative breast cancer (TNBC), where cells primed in soft environments may retain a stem‐like, drug‐resistant state even in distant tissues [25].

CD44 plays a central role in mediating these stiffness‐induced phenotypes. A well‐established marker of cancer stem cells (CSCs), CD44 functions as a signalling hub for adhesion, migration, and therapy resistance [48, 49, 50]. Isoform diversity, driven by alternative splicing, reflects the plasticity of these cells—CD44s is linked to epithelial‐to‐mesenchymal transition and tumor initiation in breast cancer [40, 41], while CD44 variant isoforms including CD44v9 have been associated with proliferation in other malignancies [51, 52, 53, 54]. CD44v9 is increasingly recognised as a marker of adaptive, stress‐responsive plasticity and microenvironmental sensing, whereas CD44s is associated with established stem‐like states. Analysing the distribution of these isoforms therefore enables discrimination between stable stemness and mechanically regulated phenotypic adaptability in heterogeneous TNBC models [36]. Both standard and variant isoforms are overexpressed in cancer and contribute to stemness, invasiveness, and resistance through interactions with matrix ligands [55, 56]. In line, CD44 isoform switching during epithelial–mesenchymal transitions have been reported in various cancer types indicating respective associations in different cell states [41]. Our findings suggest that CD44s may serve as a mechanosensitive marker of matrix‐induced stemness and could be an important target in stiffness‐driven therapeutic resistance. In contrast, CD44v9 may contribute to metastatic outgrowth in TNBC, consistent with previous reports implicating CD44 variant isoforms in metastasis across other cancer types [56].

While the field has extensively focused on how stiff matrices promote tumor aggression and spread [57, 58, 59, 60, 61, 62], only a limited number of studies have examined the pro‐tumorigenic effects of soft microenvironments. For example, Peura et al. showed that soft matrix promotes immunosuppressive pathways in TNBC [63], while matrix softness has been suggested to support plasticity and tumor‐repopulating cells in melanoma via epigenetic regulation [64]. Moreover, growing research indicates that metastases of certain cancers exhibit tissue specificity, suggesting that organ‐specific microenvironments, including their distinct ECM properties, may influence metastatic growth [65].

While some cell types prefer soft substrates for directional invasion [66], malignant cancer cells can invade across a wide stiffness range, emphasising the diverse cellular responses to substrate stiffness and their capacity to modulate molecular pathways to support functions such as proliferation or migration [67]. However, previous evidence found that certain cancer cells preferably migrated in softer substrates, prompting speculation that, as solid tumors are typically stiffer than surrounding tissues, adjacent tissue softness may naturally promote tumor cell invasion in some cancer types [68].

It is well known that ECM properties, including composition, physical and (bio)mechanical modulation, can drive cancer stemness [69], modulate tumorigenicity [43] and drive drug resistance [70]. In line with this, cells cultured in soft matrices exhibited increased resistance to the standard‐of‐care chemotherapeutic doxorubicin, accompanied by elevated ALDH1 and CD44s expression, indicative of a therapy‐resistant stem‐like fraction. Based on growing evidence that androgen receptor (AR) signalling promotes phenotypic plasticity and resistance programs in basal/TNBC [36, 71, 72, 73], and that AR pathway activity—rather than absolute expression—associates with stem‐like behavior and drug tolerance [74, 75], we incorporated the anti‐androgen enzalutamide to interrogate AR‐mediated plasticity mechanisms in this context.

Notably, while soft matrices conferred elevated resistance to single‐agent treatments, combination therapy with enzalutamide and doxorubicin significantly reduced cell viability across stiffness conditions, suggesting that targeting AR‐driven plasticity may help overcome matrix‐induced therapeutic resistance. Our work showing that mechanical softness can actively promote a therapy‐resistant stem cell fraction, thus offers a novel mechanistic link between tumor biomechanics and drug failure. Importantly, while drug exposure was the primary driver of reduced viability, matrix stiffness significantly modulated the magnitude of this response, with cells in softer matrices exhibiting higher viability than those in stiff matrices under the same treatment conditions. This highlights stiffness as an important modifier of therapeutic sensitivity rather than a sole determinant of cell viability. These results support the development of combination strategies that can target CSC populations, particularly in tumors characterized by a mechanically heterogeneous matrix at primary and metastatic sites.

Finally, this work reinforces the utility of matrix‐engineered 3D bioprinted models for high‐resolution phenotypic profiling and drug testing. Compared to 2D cultures, these platforms more accurately reproduce the structural and mechanical complexity of the tumor microenvironment. Incorporating matrix stiffness into such models could improve the predictive power of preclinical screens and support personalized treatment strategies, especially for patients with poor response to standard chemotherapy.

**FIGURE 5:**
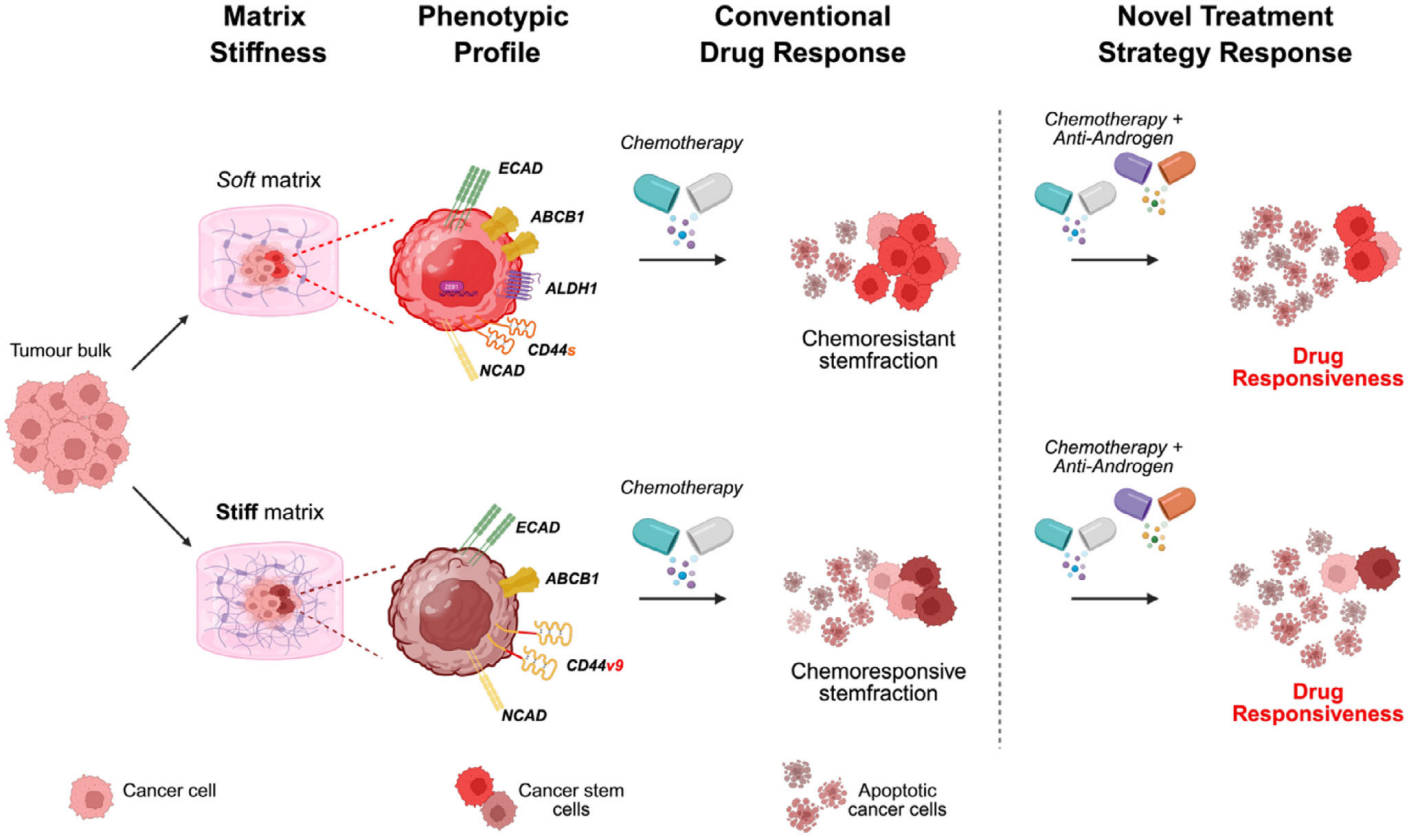
Schematic summary of matrix stiffness effects in a 3D bioprinted breast cancer model. Matrix stiffness drives phenotypic plasticity and leads to distinct drug response profiles, highlighting the role of soft matrices in promoting chemo‐resistant subpopulations. Experimental treatment strategies with combined chemotherapy (doxorubicin) and anti‐androgens (enzalutamide) are successful to target the drug resistant stem‐fraction. Our novel 3D bioprinted model system enables directed investigation of cancer progression and therapeutic resistance mechanisms.

In summary, this study demonstrates that matrix stiffness is a key regulator of cancer cell plasticity, stemness, and drug resistance in breast cancer. Using a 3D bioprinted model, we recreated defined tumor microenvironments and showed that soft matrices induce a chemoresistant stem‐like phenotype while stiffer matrices support a more proliferative, chemo‐responsive profile. These biomechanical effects on phenotype translated directly into differential drug responses, with resistance in soft matrices effectively reversed by combining chemotherapy with anti‐androgen treatment (Figure 5). Beyond these specific phenotypic outcomes, our findings establish the bioprinted 3D platform as a versatile and modular system for systematically interrogating matrix stiffness, dimensionality, and cellular phenotype in studies of cancer mechanics, plasticity, and therapeutic response. Importantly, the results obtained with the HCC38 line should be viewed as a proof‐of‐concept illustrating how this platform can reveal nuanced, context‐dependent mechanoregulation, rather than as a prescriptive model for all breast cancer subtypes. In support of this, exploratory experiments performed using the additional TNBC cell line MDA‐MB‐231 did not demonstrate the same pronounced stiffness‐dependent phenotypic changes observed in HCC38 cells, further highlighting the context‐ and cell line‐specific nature of biomechanical responses in TNBC. This model system offers a physiologically relevant platform to study tumor evolution and therapeutic response, highlighting the importance of biomechanical context in shaping tumor behavior and providing a foundation for novel strategies targeting stiffness‐induced plasticity in cancer progression and metastasis.

## 4 Conclusions

Matrix stiffness is a key determinant of breast cancer heterogeneity, shaping stemness, proliferative capacity, and drug response. Our 3D bioprinted models reveal that soft environments promote therapy‐resistant states, whereas stiff matrices sustain proliferative subsets. Importantly, combining chemotherapy with anti‐androgen treatment overcame resistance in soft matrices, suggesting that stiffness‐informed combination strategies may help target mechanically primed cancer stem‐like populations. These findings underscore the critical role of tumor mechanics in progression and therapeutic outcome.

## 5 Experimental Section

### 5.1 Cell Culture

HCC38 cells were kindly provided by A/Prof Christine Chaffer (Garvan Institute of Medical Research, Sydney, Australia) and cultured in RPMI 1640 medium (Thermo Fisher Scientific) supplemented with 10% foetal bovine serum (Bovogen) and 50 U/mL Penicillin‐Streptomycin Thermo Fisher Scientific). Cells were maintained at 37°C in a humidified chamber containing 5% CO_2_.

### 5.2 2D Microtumour Model

Two‐dimensional surfaces (Microtumors) were prepared as described in [19]. Briefly, polyacrylamide hydrogels with a stiffness of 1, 10 and 100 kPa were fabricated on aminosilanised and glutaraldehyde‐treated glass coverslips, using degassed acrylamide/bis‐acrylamide solutions polymerised between a treated coverslip and hydrophobic glass slide. After polymerisation, hydrogels were stored in water at 4°C. For functionalisation, hydrogels were treated with hydrazine monohydrate, rinsed, incubated with acetic acid, and stored overnight in Milli‐Q water. Circle patterned (⌀ 500 µm) or non‐patterned polydimethylsiloxane stamps were fabricated using photolithography. To micropattern, stamps were coated with oxidised fibronectin and gently pressed onto hydrazine‐modified hydrogels to transfer protein patterns, which were stored in water at 4°C until use. Surfaces were sterilised by 30 min exposure to UV light before cells were seeded at a concentration of 15 000 cells/mL. After 5 d with media changes every 48 h, cells were fixed with 4% paraformaldehyde (PFA, Thermo Fisher Scientific) and stored in phosphate‐buffered saline (PBS, Gibco) at 4°C until further use.

### 5.3 3D Bioprinting

Three‐dimensional bioprinting was performed using the drop‐on‐demand RASTRUM or RASTRUM Allegro bioprinters from Inventia Life Sciences [76, 77, 78]. Three‐dimensional structures were designed using the RASTRUM Cloud (Inventia Life Sciences), selecting the ‘3D Imaging Model’. Cell‐laden matrices were printed in 96‐well plates. During priming and printing of the inert base layer, cells were dissociated, centrifuged, and resuspended in 200 µL of activator solution (Inventia Life Sciences). The generation of a 3D structure was achieved by the deposition of a bioink droplet then followed by a cell‐containing droplet of activator on top, forming a covalent interaction resulting in instant gelation. The matrices used in the 3D models were based on proprietary PEG‐based bioinks developed for the RASTRUM bioprinting platform by Inventia Life Science. The physicochemical and mechanical characterisation of these bioinks, including stiffness assessment by rheological analysis, has previously been described in published studies and patent literature [76, 78]. The hydrogel stiffness conditions used in this study were 0.7 and 4.8 kPa, as defined by storage modulus measurements.

Metabolic activity as a measure of cell proliferation was assessed every 2 d for 10 d using AlamarBlue cell viability reagent (Thermo Fisher Scientific) following the manufacturer's protocol.

### 5.4 Drug Treatment

Doxorubicin hydrochloride (Sigma–Aldrich) was reconstituted in sterile ddH_2_O to prepare a stock solution of 1 mg/mL. Enzalutamide (Selleck Chemicals) was reconstituted in DMSO to prepare a stock solution of 50 mm. Bioprinted cells were cultured for 5 d, then treated with indicated drug concentrations and respective vehicle controls for 72 h. Drug response was assessed using CellTiter‐Glo 3D Cell Viability Assay (Promega) according to the manufacturer's instruction in 3 independent assays. Luminescence was measured using a CLARIOstar plate reader (BMG Labtech). Viability was calculated as percentage to vehicle‐treated control cells.

### 5.5 Immunofluorescence Staining

For 2D samples, fixed cells were permeabilized with 0.1% (v/v) Triton X‐100 for 20 min at room temperature (RT), followed by a blocking step with 1% (w/v) bovine serum albumin (BSA) in PBS. Cells were then stained with the following primary antibodies: mouse anti‐CD44 total (Cell Signaling 3570S, 1:300), mouse‐anti CD44 standard (Thermo Fisher Scientific BMS113, 1:300), mouse‐anti CD44 variant 9 (Novus Biologicals NBP2‐53204, 1:300), rabbit anti‐ZEB1 (Cell Signaling 70512S, 1:100), rabbit‐anti N‐cadherin (Cell Signaling 13116S, 1:100) and mouse‐anti E‐cadherin (Cell Signaling 14472S, 1:100) overnight at 4°C. The next day, cells were washed three times with PBS, followed by incubation with the respective secondary antibodies, goat‐anti mouse Alexa Fluor 647 (Sigma‐Aldrich SAB4600183, 1:200), donkey‐anti rabbit Alexa Fluor 555 (Sigma‐Aldrich SAB4600061, 1:200), Atto‐488 Phalloidin (Sigma‐Aldrich 49409–10NMOL, 1:200) and DAPI (Thermo Fisher Scientific 62248, 1:1000) for 1 h at RT, followed by 3 washing steps with PBS and a final wash in ddH_2_O. Coverslips with cells were mounted with ProLong Glass Antifade Mounting Media (Thermo Fisher Scientific) on a glass slide and cured for 24 h at RT in the dark.

For 3D samples, tumoroids within the 3D bioprinted matrices were fixed with 4% PFA for 24 h at RT, then permeabilised with 2% (v/v) Triton X‐100 overnight at RT. The next day, samples were washed three times in PBS every 2 h, then stained with primary antibodies as described above. Additional primary antibodies were used: mouse‐anti aldehyde dehydrogenase‐1 (Thermo Fisher Scientific PA5‐32127, 1:200), rabbit‐anti ABCB1 (Cell Signaling 12683S), 1:200), mouse‐anti CD133 (Thermo Fosher Scientific PA538014, 1:100), rabbit‐anti‐Ki67 (Invitrogen, 1:100). Samples were incubated for 72 h at RT on a shaker, followed by four PBS washes every 2 h, a final washing step overnight and incubation with secondary antibodies and stains described above for 48 h at RT on a shaker. Four PBS washes every 2 h, followed by a final washing step overnight were performed before equilibrating the samples with a refractive‐index matching solution (optical clearing) containing 60% (v/v) glycerol and 2.5 m fructose overnight at 4°C in the dark.

### 5.6 Confocal Laser‐Scanning Microscopy

Fluorescence images were acquired using a confocal laser scanning microscope (Zeiss LSM 800; KGLMF, MWAC, UNSW Sydney) equipped with a Plan‐Apochromat 20×/0.8 M27 air objective and Zen Blue imaging software (Zeiss, Germany). Alexa Fluor 647, Alexa Fluor 555, Atto‐488 Phalloidin and DAPI were excited with 640 nm, 561 nm, 488 nm and 405 nm laser lines, respectively. Fluorophores were detected as follows: Alexa Fluor 647 (Ex 653 nm/Em 668 nm; detection 656–700 nm), Alexa Fluor 555 (Ex 553 nm/Em 568 nm; detection 555–645 nm), Atto‐488 Phalloidin (Ex 500 nm/Em 525 nm; detection 410–555 nm), and DAPI (Ex 353 nm/Em 465 nm; detection 410–555 nm). Images were acquired at 1724 × 1724 pixels (0.12 µm per pixel) with a pixel dwell time of 1.22 µs and z‐stack slices of 0.5 µm. All immunofluorescence quantification was performed using data from *n* = 3 independent biological experiments, with images acquired and analysed from 4–6 distinct regions of interest (ROIs) per condition in each experiment.

### 5.7 Quantification and Statistical Analysis

Unless otherwise noted, all experimental results are pooled from at least three independent experiments ± standard error of the mean (SEM). Watershed segmentation and quantification of 2D samples was performed with a customised MATLAB GUI (MathWorks, available upon request); segmentation of cells and quantification of immunostained 3D bioprinted samples was performed using IMARIS image analysis software (Oxford Instruments). The segmentation results from Imaris were exported into Excel that were further imported into a custom‐built MATLAB script (MathWorks) to quantify the morphological metrics of 3D bioprinted samples before and after drug treatment.

For data with normal distribution, one‐way analysis of variance (ANOVA) with Tukey's post hoc test for multiple comparisons was used. For data with non‐normal distribution, Kruskal‐Wallis H test with Dunn's post hoc test for multiple comparisons was used. All statistical tests were performed using Prism version 10 (GraphPad). Statistical details are provided in the figure legends. Error bars represent the standard error of the mean (SEM). *p*‐value is reported for statistical significance. Comparisons between samples were considered to be statistically significant if the *p*‐value was *p < 0.05, **p < 0.01, ***p < 0.001, *****p < 0.0001.

## Author Contributions

C.K. and K.A.K. designed the study, and K.A.K. and J.G oversaw conceptualization. C.K. performed all experiments and prepared the manuscript. E.P. contributed to data analysis, and S.P. contributed to the 3D bioprinting experiments. K.A.K. and J.G. were responsible for financial support. All authors contributed to the manuscript editing.

## Conflicts of Interest

The authors declare no conflicts of interest.

## Supporting information




**Supporting File**: adhm71371‐sup‐0001‐SuppMat.docx.

## Data Availability

The data that support the findings of this study are available from the corresponding author upon reasonable request.
