# Supplementary material for: Matrix Stiffness Directs Stemness Signatures in Breast Cancer
Source: Adv Healthc Mater. 2026 Jun 22;15(28):e71371. doi: 10.1002/adhm.71371 (PMC13410852; doi:10.1002/adhm.71371)
Supplement: Supplementary file 1 — Supporting File: adhm71371‐sup‐0001‐SuppMat.docx. [file ADHM-15-0-s001.docx]

**SUPPORTING INFORMATION**

**Matrix stiffness directs stemness signatures in breast cancer**

Chantal Kopecky^1^, Elvis Pandzic^2^, Sean Porazinski^3^, Justin Gooding^1^, Kristopher A Kilian^1,4^

*^1^ School of Chemistry, Australian Centre for NanoMedicine, Faculty of Science, UNSW Sydney, Australia*

*^2^ Katharina Gaus Light Microscopy Facility, Mark Wainwright Analytical Centre, UNSW Sydney, Australia*

*^3^ Inventia Life Science, Sydney, Australia*

1. *School of Material Sciences & Engineering, Faculty of Science, UNSW Sydney, Australia*

Correspondence to Kristopher Kilian (k.kilian@unsw.edu.au) and Justin Gooding (justin.gooding@unsw.edu.au)

**SUPPLEMENTARY FIGURES**


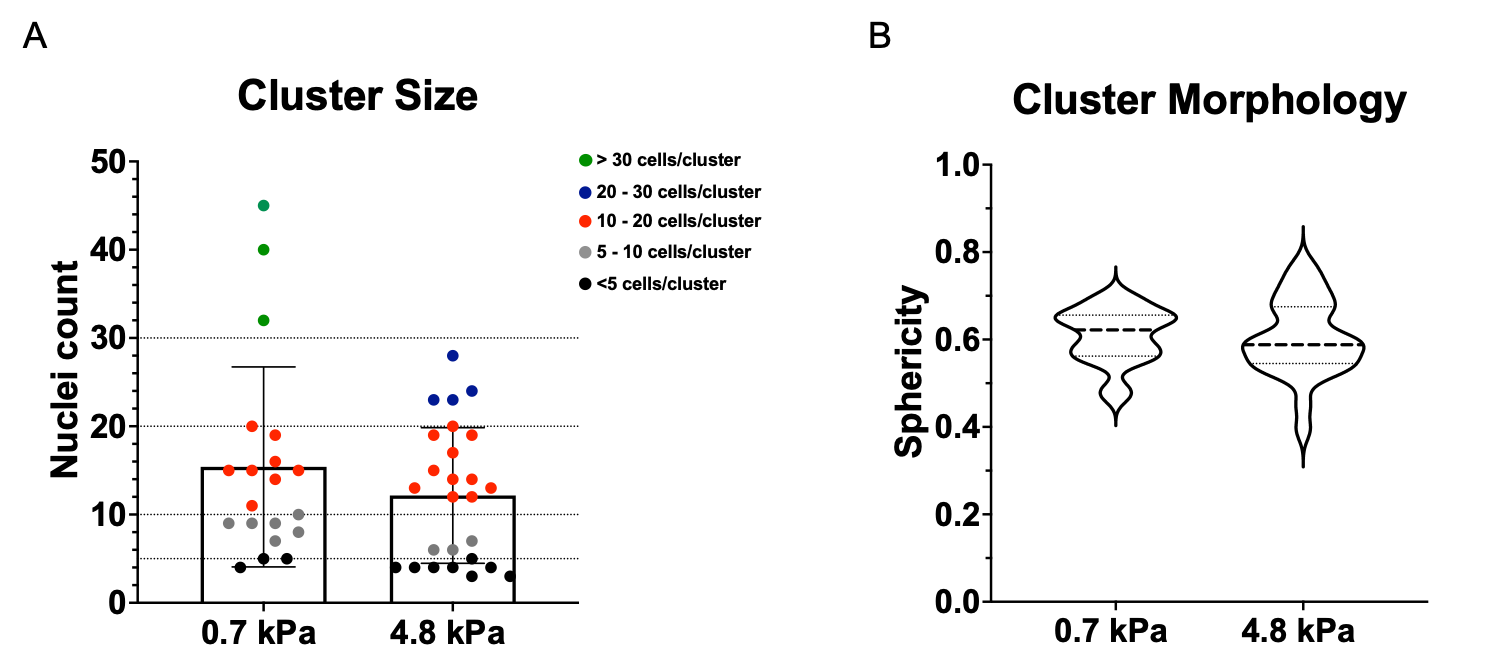


**Supplemental Figure 1.** Matrix stiffness and morphological characteristics of 3D bioprinted breast cancer models. **A)** Cluster sizes of HCC38 tumouroids cultured for 10 d in soft (0.7 kPa) and stiff (4.8 kPa) matrices was assessed by quantifying nuclei counts of immunostained samples. Cluster sizes were displayed in margins of indicated number of cells/cluster. **B)** Cluster morphology was assessed by quantifying the sphericity of 3D rendered nuclei from immunostained samples and displayed as sphericity.


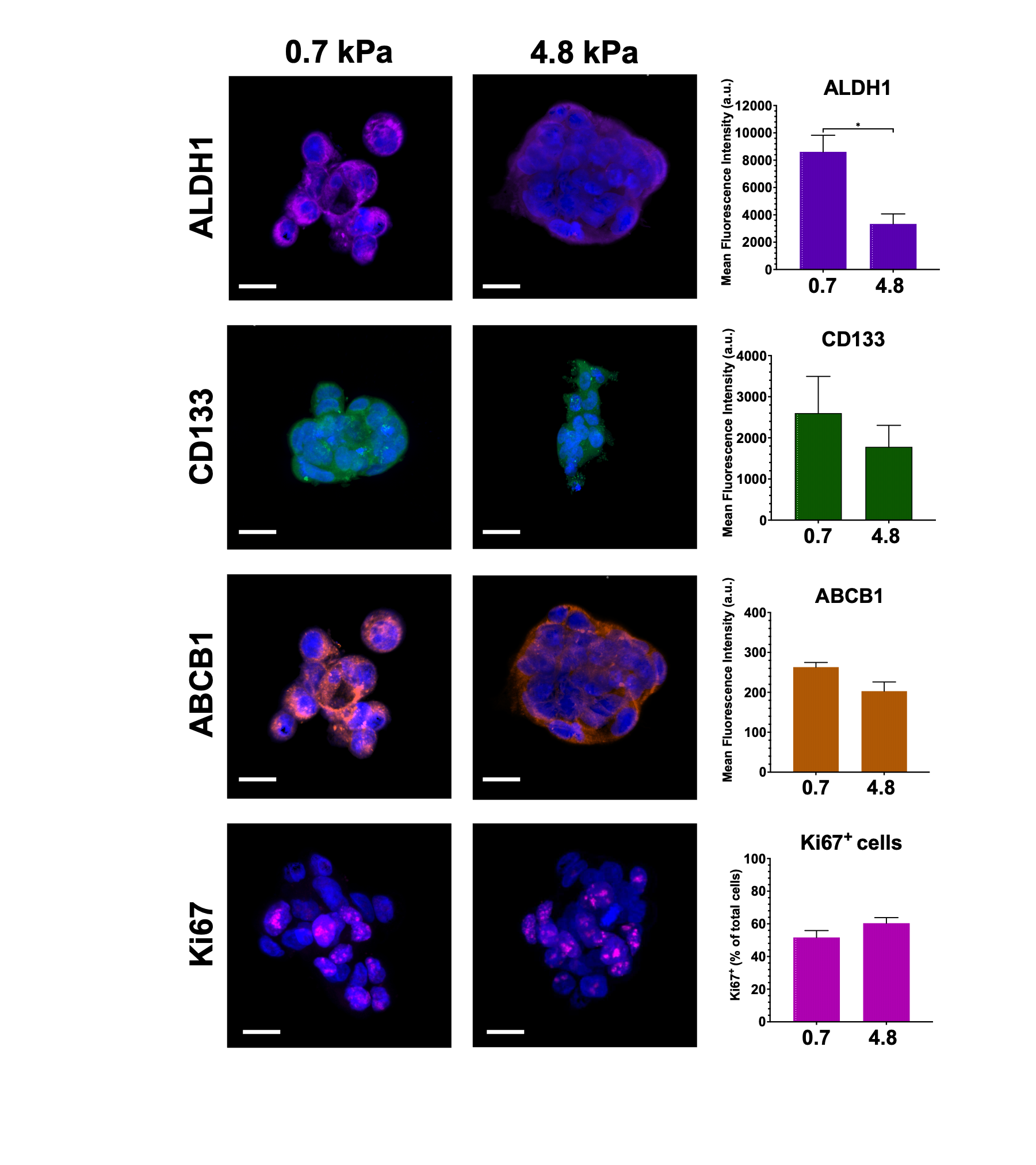


**Supplemental Figure 2.** Soft matrix induces a specific phenotypic plasticity profile in 3D bioprinted breast cancer models. HCC38 tumouroids were immunostained for indicated plasticity markers after encapsulation for 10 d in soft (0.7 kPa) and stiff (4.8 kPa) matrices.

*
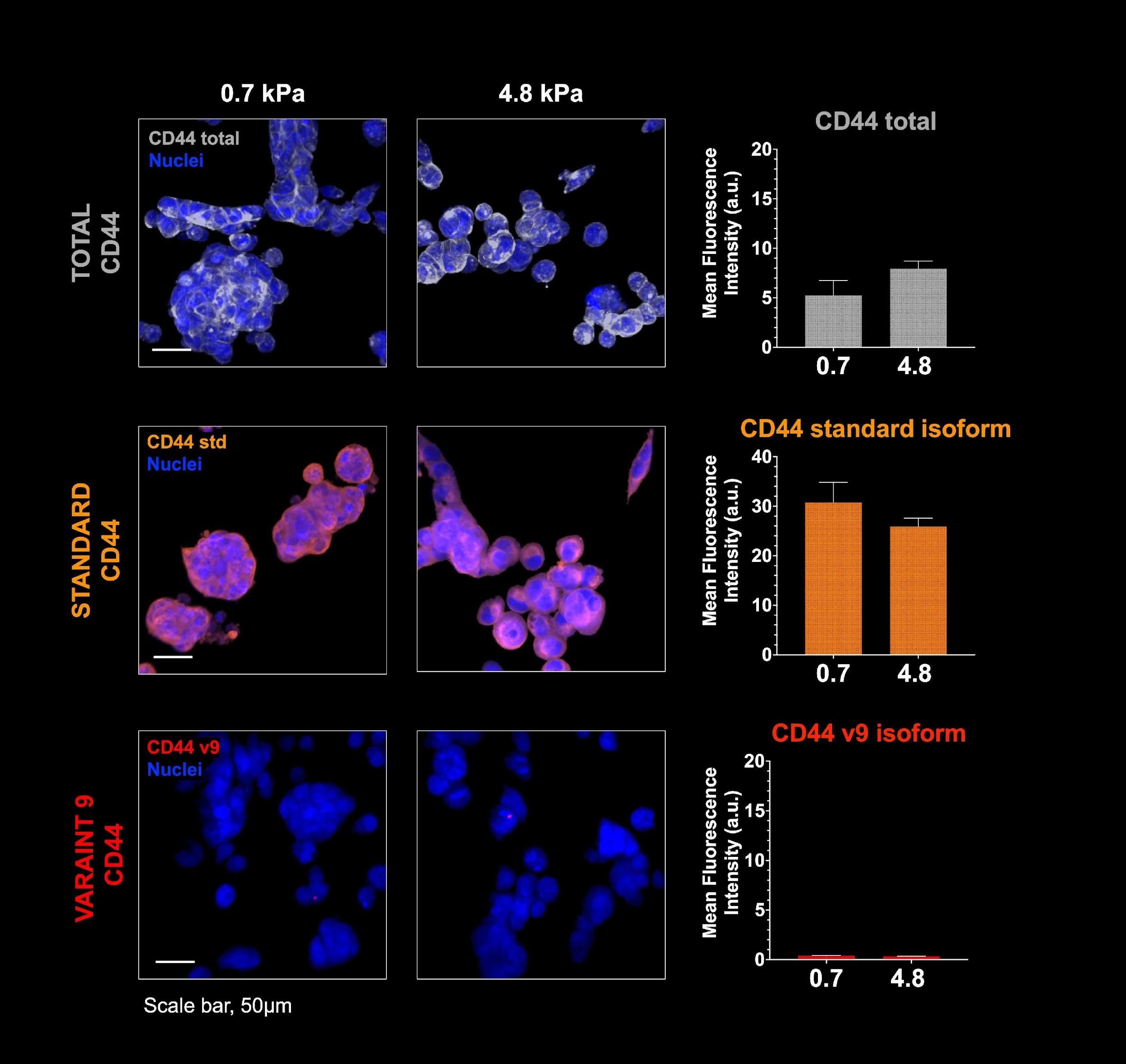
*

*
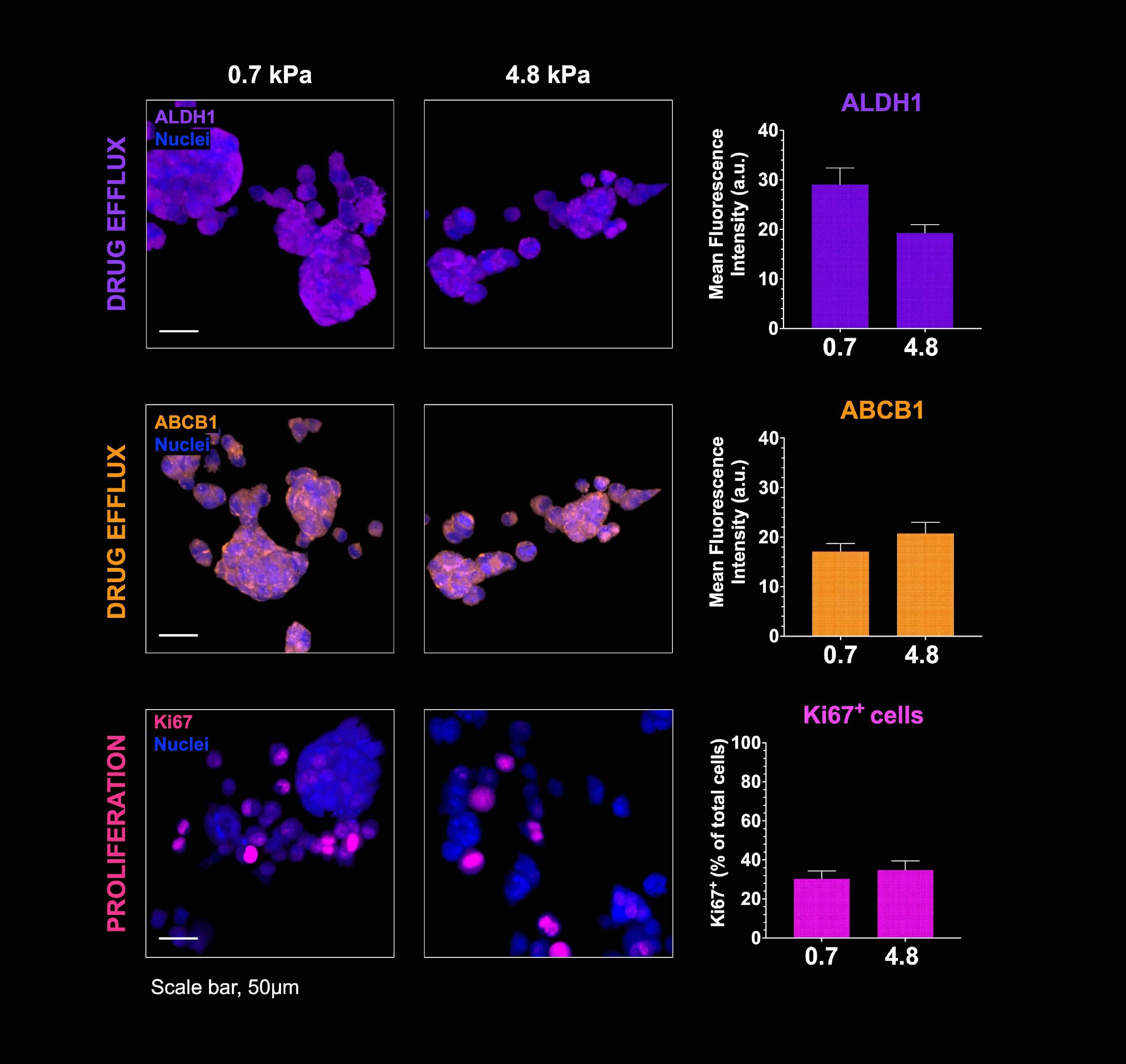
*

*
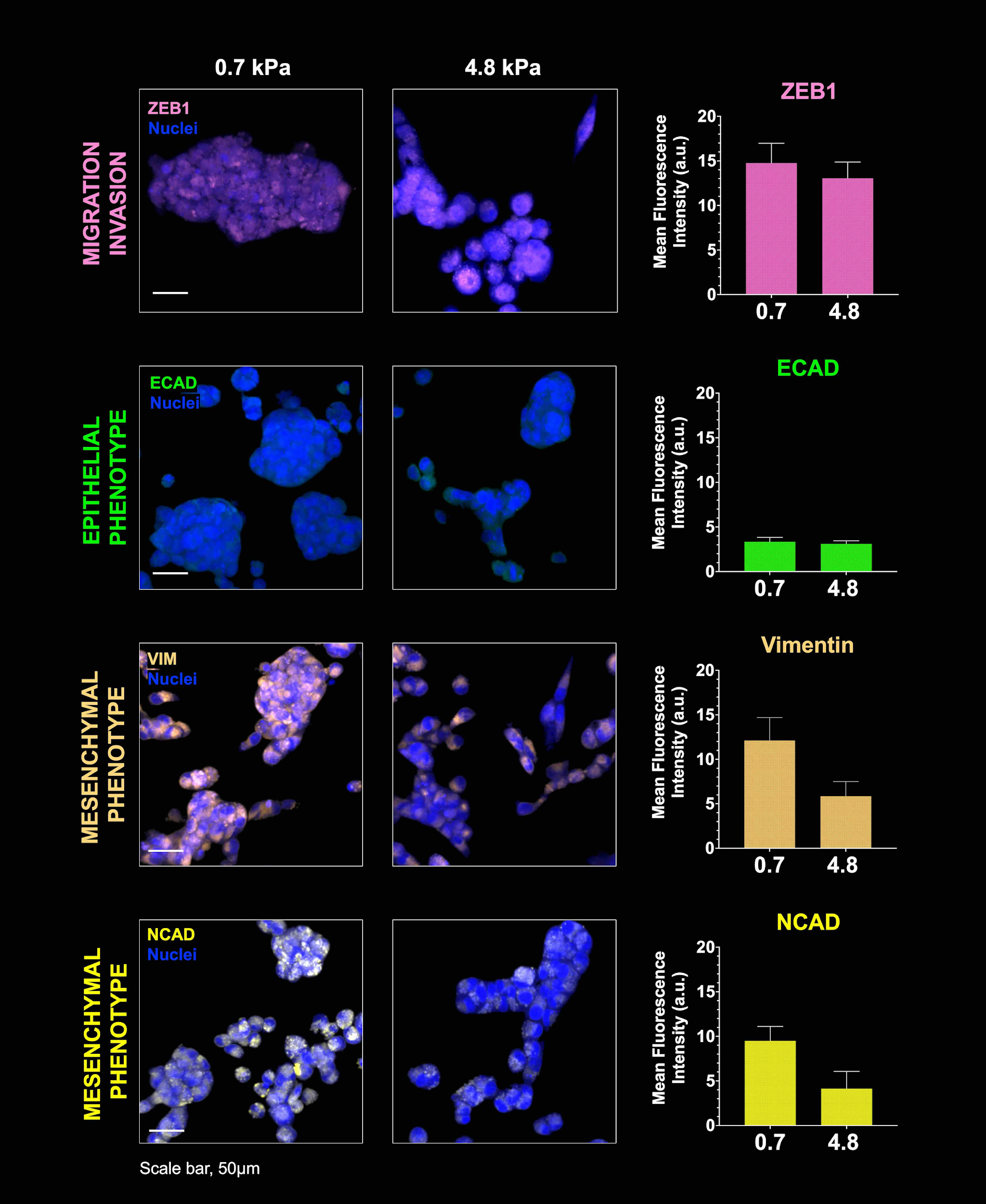
*

**Supplementary Figure 3.** Phenotypic characterisation of 3D bioprinted breast cancer models after 10 d of culture. Representative immunofluorescence images of MDA-MB-231 tumoroids cultured in soft (0.7 kPa) and stiff (4.8 kPa) matrices, analysed for stemness (CD44 isoforms), plasticity (ALDH1, ABCB1, Ki67), and migration-related markers (ZEB1, E-cadherin, Vimentin, N-cadherin). Immunofluorescence quantification was performed using data from n = 1 exploratory experiment, with images acquired and analysed from 3-4 distinct regions of interest (ROIs) per condition.


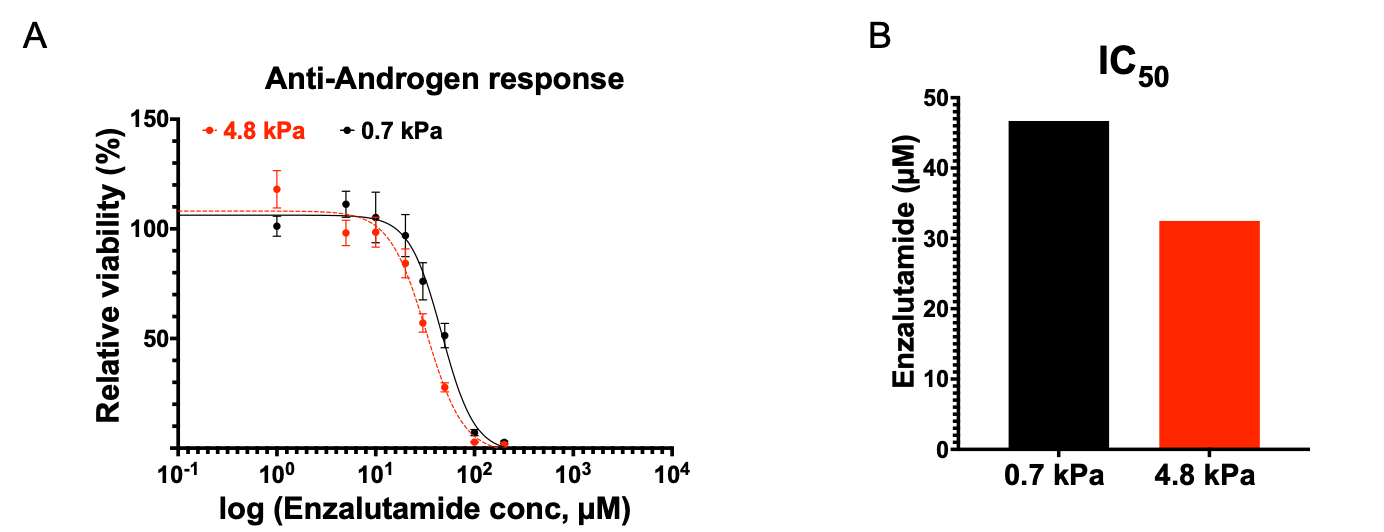


**Supplemental Figure 4.** Matrix stiffness elicits a distinct drug response to anti-androgen therapy. **A)** Dose-response curves of HCC38 tumouroids encapsulated for 5d in soft (0.7 kPa) and stiff (4.8 kPa) matrices treated with various enzalutamide concentrations for 72 h, followed by viability assays. **B)** Half maximal inhibitory concentrations (IC_50_) for different stiffness conditions.

_
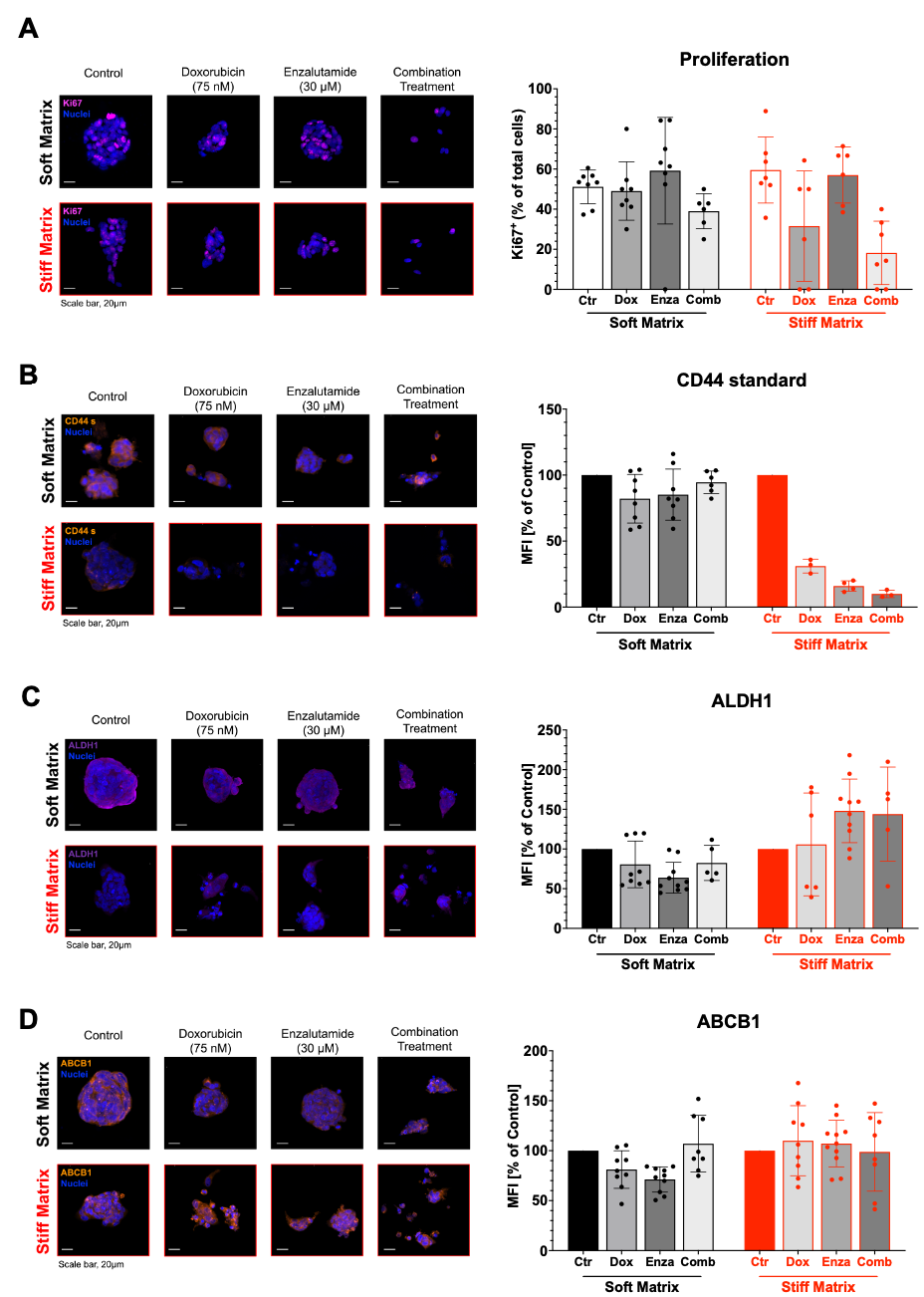
_

**Supplemental Figure 5.** Immunostaining of indicated **A)** proliferation and **B-D)** stemness markers and drug efflux transporters after treatments to assess the phenotypic changes.

Ctr, untreated control; Dox, doxorubicin; Enza, enzalutamide; Comb, combination treatment.
